# Abnormal expression of galectins and their correlation with fibrogenesis in adenomyosis

**DOI:** 10.1080/19336918.2026.2658283

**Published:** 2026-05-20

**Authors:** Yuan Zhou, Mengdong Cao, Zhenzhen Wang, Yaonan Yu, Han Yu, Xue Shen

**Affiliations:** a Department of Gynaecology and Obstetrics, Xiangyang No.1 People’s Hospital Graduate Joint Training Base, School of Medicine, Wuhan University of Science and Technology, Xiangyang, P.R. China; b Department of Gynaecology and Obstetrics, Xiangyang No.1 People’s Hospital, Hubei University of Medicine, Xiangyang, P.R. China; c Department of Pathology department, Xiangyang No.1 People’s Hos pital, Hubei University of Medicine, Xiangyang, P.R. China

**Keywords:** Adenomyosis, galectin, dysmenorrhea, fibrosis, uterine volume

## Abstract

This cross-sectional study investigated galectin-1, −3, and −9 in adenomyosis. Analysis of hysterectomy tissues from premenopausal women via western blot, qPCR, immunohistochemistry, and Masson’s staining revealed significantly elevated galectin levels compared to controls. Galectin expression positively correlated with fibrosis severity (*r* = 0.476, 0.925, 0.563, all *p* < .05), which in turn moderately correlated with dysmenorrhea (VAS score) and uterine enlargement. Findings indicate that galectins are implicated in adenomyosis-related fibrosis and pain, suggesting their potential as therapeutic targets.

## Introduction

Adenomyosis (AM) is a prevalent gynecological disease characterized by the ectopic invasion and proliferation of endometrial glands and stroma into the myometrium, resulting in focal or diffuse uterine enlargement [1]. The incidence of adenomyosis has steadily increased in recent years, with a notable trend toward younger affected populations [2]. Although classified as a benign condition, adenomyosis exhibits pathological features and biological behaviors reminiscent of malignant tumors. Emerging evidence indicates that fibrosis is not only implicated in the pathogenesis of various tumors [3], but also constitutes a key mechanisms underlying the development and progression of adenomyosis [4]. Fibrosis represents a hallmark of aberrant tissue repair in response to sustained or repeated injury [5]. Adenomyosis can thus be conceptualized as a ‘wound’ undergoing recurrent injury-repair cycles. This process ultimately drives fibrotic remodeling through mechanisms such as epithelial-mesenchymal transition (EMT), fibroblast-to-myofibroblast transdifferentiation, and smooth muscle metaplasia [6]. This fibrotic cascade is mediated by a complex interplay of cytokines, hormones, and growth factors. These mediators not only perpetuate tissue fibrosis but also contribute to neurogenic dysregulation and heightened pain sensitivity, thereby exacerbating dysmenorrhea [7].

Galectins (Gals) are a family of small, evolutionarily conserved endogenous lectins defined by their affinity for β-galactoside-containing glycans. Comprising 12 members, these proteins are ubiquitously expressed in human tissues and organs [8]. Galectins play pivotal roles in diverse physiological and pathological processes – including inflammation, tumorigenesis, immune regulation, and reproduction – making them promising therapeutic targets for cancer and other diseases [9]. Accumulating evidence has established that Gals serve as critical regulators in the pathophysiology of various gynecological diseases. In gynecological malignancies, Gals are implicated in tumor cell proliferation, invasion, immune evasion, and the modulation of tumor-associated fibrosis [10,11]. In endometriosis, these molecules facilitate the adhesion, proliferation, and collagen deposition of ectopic endometrial cells, thereby contributing to lesion development, fibrotic progression, and pelvic adhesions [12]. Adenomyosis shares key pathological features with endometriosis, including ectopic endometrial infiltration, smooth muscle hyperplasia, chronic inflammation, and extensive tissue fibrosis [13]. Moreover, it exhibits persistent inflammation and stromal remodeling analogous to the tumor microenvironment. Based on these pathological commonalities, we hypothesized that Gals may also play a significant role in the pathogenesis and progression of adenomyosis.

Among the galectin family, Galectin-1 (Gal-1), Galectin-3 (Gal-3), and Galectin-9 (Gal-9) have been most closely associated with the regulation of inflammatory responses and fibrotic processes [14]. Accordingly, we selected these three members as candidate molecules to systematically investigate their expression profiles and clinical relevance in adenomyosis. Specifically, we examined the expression profiles of prototype Gal-1, chimera-type Gal-3, and tandem-repeat-type Gal-9 in adenomyotic lesions to elucidate their potential roles in disease pathogenesis and progression. Furthermore, we performed comprehensive correlation analyses between galectin expression levels and both the degree of myometrial fibrosis and the severity of dysmenorrhea in adenomyosis patients. These findings provide new insights into the molecular mechanisms underlying adenomyosis-associated dysmenorrhea and may inform the development of targeted therapeutic strategies.

## Materials and methods

### Patients and specimens

The study was approved by the Ethics Committee of Xiangyang No.1 People’s Hospital (Approval No.: 2021-TH-068), and all participants provided written informed consent. Uterine samples were collected at Xiangyang No.1 People’s Hospital between August 2023 and October 2024 from two patient groups. The control group consisted of 30 premenopausal women who underwent hysterectomy for cervical intraepithelial neoplasia grade II/III, early-stage cervical cancer, or ovarian cancer. Clinical examination revealed regular menstrual cycles, no signs of AM, and no history of primary dysmenorrhea in these patients. The study group included 30 premenopausal women diagnosed with adenomyosis based on histopathological examination, with the diagnostic criterion defined as endometrial glands penetrating > 2.5 mm below the endometrial-myometrial junction. During the operation, tissue specimens with a size of 0.5 cm × 0.5 cm × 0.5 cm were excised from the uterine myometrium. Subsequent to the operation, all specimens were sent for pathological examination to confirm their compliance with the research requirements.

All patients were premenopausal women. Exclusion criteria comprised: endometrial polyps, hyperplasia, or cancer; uterine myomas; other significant endometrial or myometrial pathologies; concurrent endocrine, immune, or metabolic disorders; intrauterine device (IUD) placement; or hormone therapy within 3 months prior to surgery. The mean ages of the control and AM groups were 47.4 ± 3.9 years and 47.8 ± 5.0 years, respectively. No significant differences were observed in age (*p* = .518) or body mass index (BMI, *p* = .399) between groups. Patient baseline characteristics are summarized in Table 1. Further Logistic regression analysis revealed that the VAS score for dysmenorrhea (*p* = .041, OR = 4.312, 95% *CI*: 1.061–17.530) was an independent risk factor for the occurrence of adenomyosis. See Table 2 for details.

**Table 1.** Characteristics of women in the adenomyosis and control groups and significant differences between the two groups.

Characteristic	Control group (*n* = 30)	Adenomyosis group (*n* = 30)	*P* value
Age, years, median (IQR)	48 (5.25)	48 (6)	0.518
BMI, kg/m^2^, mean (SD)	24.07 (2.92)	24.74 (3.17)	0.399
Gravidity, median (IQR)	2 (3)	2 (2)	0.132
Parity, median (IQR)	1 (1)	1 (1)	0.667
Menstrual length, mean (SD)	5.77 (1.72)	8.07 (2.59)	0.000
VAS, mean (SD)	2.07 (1.05)	6.00 (1.95)	0.000
Hemoglobin (g/l), mean (SD)	123.13 (14.56)	107.40 (26.31)	0.006
CA125, U/ml, median (IQR)	23.97 (29.83)	61.95 (83.58)	0.011
Uterine volume, cm^3^,mean (SD)	81.03 (33.29)	267.64 (89.90)	0.000
Marital status			1.000
Married	26 (86.67%)	27 (90%)	
other	4 (13.33%)	3 (10%)	
History of uterine operation			0.796
Yes	15 (50%)	16 (53.33%)	
No	15 (50%)	14 (46.67%)

**Table 2.** Univariate and multivariate analysis of factors associated with adenomyosis.

Factors	Univariate analysis		Multivariate analysis	
	*OR* (95%*CI*)	*P*	*OR* (95%*CI*)	*P*
Menstrual length	1.727 (1.223 ~ 2.438)	0.002	1.103 (0.458 ~ 2.659)	0.827
VAS	4.426 (2.164 ~ 9.050)	0.000	4.312 (1.061 ~ 17.530)	0.041
Hemoglobin	0.964 (0.937 ~ 0.992)	0.011	0.958 (0.874 ~ 1.049)	0.353
Uterine volume	1.043 (1.019 ~ 1.067)	0.000	1.036 (1.008 ~ 1.065)	0.011
CA125	1.009 (1.000 ~ 1.018)	0.049	0.996 (0.976 ~ 1.016)	0.671

### Pain evaluation

A visual analogue score (VAS; 0–10, where 0 = no pain and 10 = maximum pain) was used before surgery to assess the severity of dysmenorrhea. Scores were categorized as mild (0–3), moderate (4–6), or severe (7–10). Among the AM patients, seventeen presented with mild-to-moderate dysmenorrhea and thirteen with severe dysmenorrhea.

### Uterine volume

Uterine volume was measured preoperatively by transvaginal using the ovoid formula: uterine volume = D1 × D2 × D3 × 0.52 [15], where D1, D2, and D3 represent the vertical, transverse, and anteroposterior diameters, respectively.

### Immunohistochemistry

All tissue samples were sectioned at a thickness of 4-μm. The paraffin-embedded sections were deparaffinized using an environmentally friendly dewaxing agent followed by a graded ethanol series for rehydration. For antigen retrieval, sections were boiled in citrate buffer (10 mmol/L, pH 6.0) for 0.5 hours. Subsequently, endogenous peroxidase activity was blocked by incubating the sections in 3% hydrogen peroxide for 25 minutes. The sections were then blocked with 3% bovine serum albumin (BSA; Servicebio, Wuhan, China) at room temperature for 30 minutes, followed by overnight incubation at 4°C with rabbit-derived primary antibodies against Gal-1, Gal-3, or Gal-9 (Catalog Nos.: 11,858–1-AP, GB151145, 17,938–1-AP; Cell Signaling Technology, Danvers, MA, USA). After washing with PBS, the sections were incubated at room temperature for 50 minutes with horseradish peroxidase (HRP)-conjugated goat anti-rabbit secondary antibody (Servicebio, Wuhan, China). Color development was performed using DAB under microscopic observation, and nuclei were counterstained with hematoxylin. Finally, sections were dehydrated through a graded ethanol series, cleared in xylene, mounted with neutral balsam, and observed under a light microscope for image acquisition. Immunohistochemical staining results were semi-quantitatively analyzed using Image-Pro Plus 6.0 software (Media Cybernetics, Rockville, MD, USA) by researchers who were blind to patient group assignments. For each target protein, ten fields of view were randomly selected from each section for analysis, and the average values were used for subsequent statistical comparisons.

### Protein extraction and Western blot

Protein extraction was first performed: suspension cells were collected by centrifugation and lysed by vortexing in RIPA buffer containing protease inhibitors (radioimmunoprecipitation assay: phenylmethanesulfonyl fluoride, 100:1); adherent cells were washed with PBS and directly collected by scraping in lysis buffer; tissue samples were ground in liquid nitrogen before lysis. All lysates were incubated on ice for 30 minutes, then centrifuged at 12,000 rpm and 4°C for 10 minutes to collect the supernatant as total protein. Protein concentration was measured using the BCA method, after which samples were mixed with reducing loading buffer and denatured at 95°C for 10 minutes for later use. SDS-PAGE electrophoresis (10% gradient gels; Criterion Gel System) (Bio-Rad, Hercules, CA) was then conducted: gels were prepared following standard protocols, samples were loaded, and electrophoresis was performed at a constant voltage of 200 V for approximately 30 minutes. After electrophoresis, proteins were transferred onto a PVDF membrane activated with methanol using the wet transfer method (constant current of 300 mA, 30 minutes). Following transfer, immunodetection was carried out: the membrane was blocked with 5% skim milk at room temperature for 30 minutes, incubated with diluted primary antibodies at 4°C overnight, washed with TBST, and then incubated with HRP-conjugated secondary antibodies (1:5000 dilution) at room temperature for 30 minutes. After washing, protein bands were visualized using ECL Iuminescent solution (Servicebio, Wuhan, China). Finally, Blots were imaged on a Chemiluminescence apparatus (Servicebio, Wuhan, China) and quantified using densitometry.

### RNA isolation and real-time quantitative reverse transcription polymerase chain reaction

Total RNA was extracted from each sample with a NanoDrop 2000 Spectrophotometer. The SweScript All-in-One RT SuperMix for qPCR (G3337) (Servicebio, Wuhan, China) was used to synthesize cDNA from total RNA per sample. The primers used in this study were designed by Servicebio, Wuhan, China. The sequences are presented in Table 3. Polymerase chain reactions were carried out on a fluorescent quantitative PCR apparatus. The reaction began at 95°C for 30 s for initial denaturation, followed by 40 cycles of 15 s at 95°C and 30 s at 60°C. The temperature rose gradually, and the fluorescence signal was collected every time the temperature rose 0.5°C. According to research by Chapman and Waldenström [16], ACTIN was used, and the geometric mean of reference gene expression levels was used for normalization. The 2−ΔΔCT method was used to analyze the relative gene expression as follows [17].

**Table 3.** Specific primers used in quantitative reverse transcription polymerase chain reaction analysis.

Gene		Sequence (5′-3′)
Galectin-1	Forward	5’-TTCAACCCTCGCTTCAACG-3’
	Reverse	5’-GCGGTTGGGGAACTTGAAT-3’
Galectin-3	Forward	5’-GCCACTGATTGTGCCTTATAACC-3’
	Reverse	5’-AAAACCGACTGTCTTTCTTCCC-3’
Galectin-9	Forward	5’-CTCTACAAAGGACTTCCTAGTGGGT-3’
	Reverse	5’-TCACAGCAAACCTGGTTCCACT-3’
GAPDH	Forward	5’-GGAAGCTTGTCATCAATGGAAATC-3’
	Reverse	5’-TGATGACCCTTTTGGCTCCC-3’

Fold change = 2−ΔΔCT.

2−ΔΔC *T* = ([CT gene of interest- CT RG] sample A – [CT gene of interest- CT RG] sample B).

### Masson staining

Masson’s trichrome staining was used to detect collagen fibers in tissue samples. Tissue sections (4 μm, paraffin-embedded) were deparaffinized in xylene, rehydrated in a graded alcohol series, and then soaked in Bouin’s solution at 37°C for 2 h. Bouin’s solution was made with 75 mL of saturated picric acid, 25 mL of 10% formalin solution (v/v), and 5 mL of acetic acid. Tissue sections were stained using a Masson’s staining kit (Servicebio, Wuhan, China). Following Masson staining, the sections were observed and images were captured using an upright optical microscope (Nikon, Japan). Collagen fibers appeared blue, smooth muscle fibers red, and red blood cells also red [15]. The relative content of collagen fibers was determined by calculating the ratio of positively stained tissue area to the total tissue area using Image-Pro Plus 6.0 software.

## Statistical analysis

SPSS Statistics for Windows, Version 19.0. was used for statistical analysis of the data. The measurement data meeting the normal distribution were represented by mean ± standard deviation $\left(\overset{ˉ}{X}\pm S\right)$, and an independent sample t-test was used. Measurement data that did not satisfy the normal distribution were represented by quartile M (*P*25, *P*75), and the Mann-Whitney U test was used. The counting data were expressed by frequency (n) and rate (%), and the χ^2^ test was used. Logistic regression analysis was performed to identify factors independently associated with the presence of adenomyosis. Variables showing significant differences between the two groups in univariate analysis were entered into a multivariate logistic regression model. Results are presented as odds ratios (ORs) with 95% confidence intervals (CIs).The correlations among Gal-1/Gal-3/Gal-9 expression, fibrosis, and the severity of dysmenorrhea were assessed using Pearson’s correlation and Spearman’s correlation. All test results were statistically significant with *p* < .05.

## Results

### Gal-1/Gal-3/Gal-9 protein expression levels in the myometrium of adenomyosis and the control group

**Figure 1.**
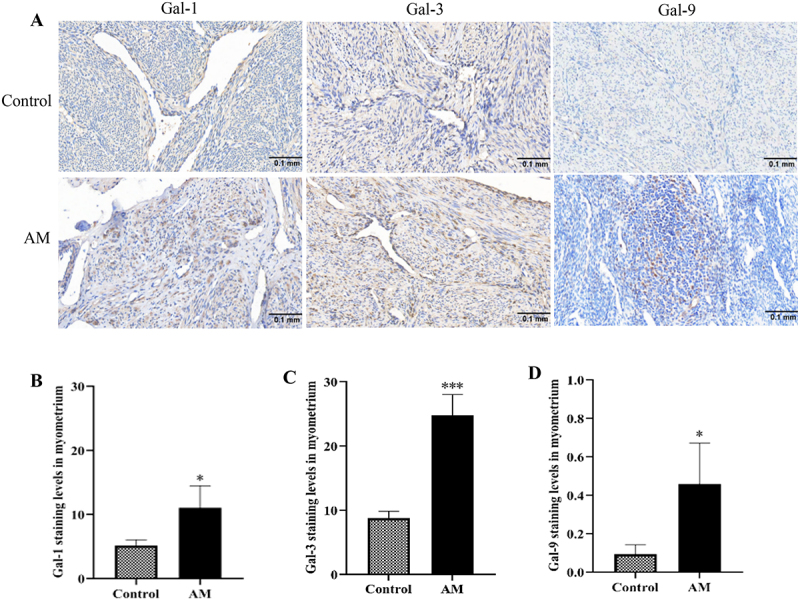
The expression of gal-1, gal-3, and gal-9 by immunohistochemical staining in the adenomyosis group (am) and the control group (×100).

**Figure 2.**
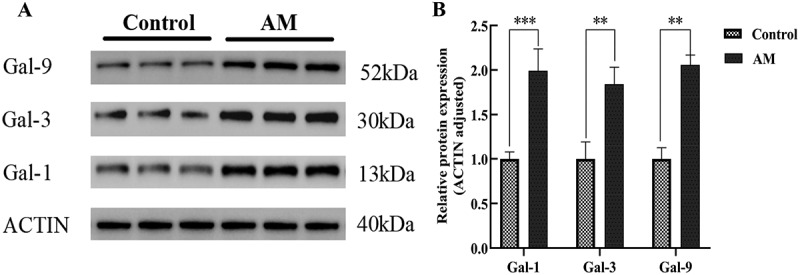
The expression of gal-1, gal-3, and gal-9 in the adenomyosis group (am group) and the control group.

Immunohistochemical analysis indicated that Gal-1, Gal-3, and Gal-9 were predominantly localized in the cytoplasm, exhibiting a color range from light yellow to brown. Quantitative analysis of the experimental data revealed that the expression levels of Gal-1, Gal-3, and Gal-9 proteins were significantly elevated in the AM group compared to the control group’s myometrium, with statistical significance (*p* < .05), as illustrated in Figure 1. To corroborate these findings, Western blot analyses were performed to assess the expression of Gal-1, Gal-3, and Gal-9 proteins (Figure 2). Consistent with the immunohistochemical results, Western blot data demonstrated a significant upregulation of Gal-1, Gal-3, and Gal-9 protein levels in the myometrium of AM patients relative to normal myometrium (*p* < .05). These findings imply that Gal-1, Gal-3, and Gal-9 May play a critical role in the pathogenesis of AM.

(A) Immunohistochemical images of the expression of Gal-1, Gal-3, and Gal-9 in the AM group and the control group (B) Comparison of the expression of Gal-1 in the myometrium of the two groups of patients; (C) Comparison of the expression of Gal-3 in the myometrium of the two groups of patients; (D) Comparison of the expression of Gal-9 in the myometrium of the two groups of patients. All micrographs are at 100 × magnification. Scale bars represent 0.1mm. * *p* < 0.05, ** *p* < 0.01, *** *p* < 0.001.

(A) Western blot analysis of Gal-1, Gal-3, and Gal-9 expression in the myometrium of patients in the control and AM groups. ACTIN was used as a sample loading control. (B) The relative densities of Gal-1, Gal-3, and Gal-9 were normalized to ACTIN in the myometrium of patients in the control and AM groups. * *p* <0.05, ** *p* <0.01, *** *p* < 0.001.

### Gal-1/Gal-3/Gal-9 mRNA expression levels in the myometrium of adenomyosis and the control group

**Figure 3.**
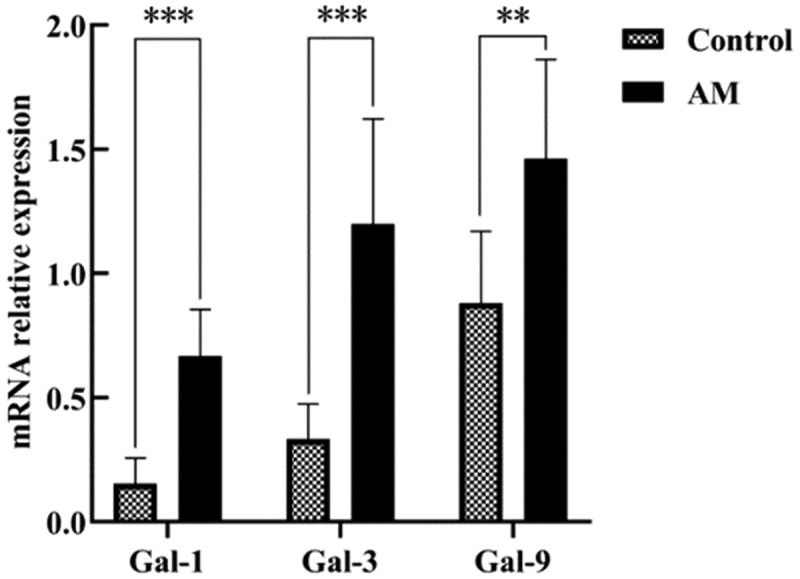
Comparison of the expressions of gal-1 mRNA, gal-3 mRNA, and gal-9 mRNA in the am group and the control group. *p < .05, **p < .01, ***p < .001.

To further assess the expression levels of Gal-1, Gal-3, and Gal-9 in patients with AM compared to controls, real-time quantitative polymerase chain reaction was employed to measure the mRNA expression of these galectins. The results corroborated previous findings, demonstrating that the mRNA levels of Gal-1, Gal-3, and Gal-9 were significantly elevated in the myometrium of patients with AM relative to the control group (Figure 3).

### Fibrosis characteristics in adenomyotic lesions

**Figure 4.**
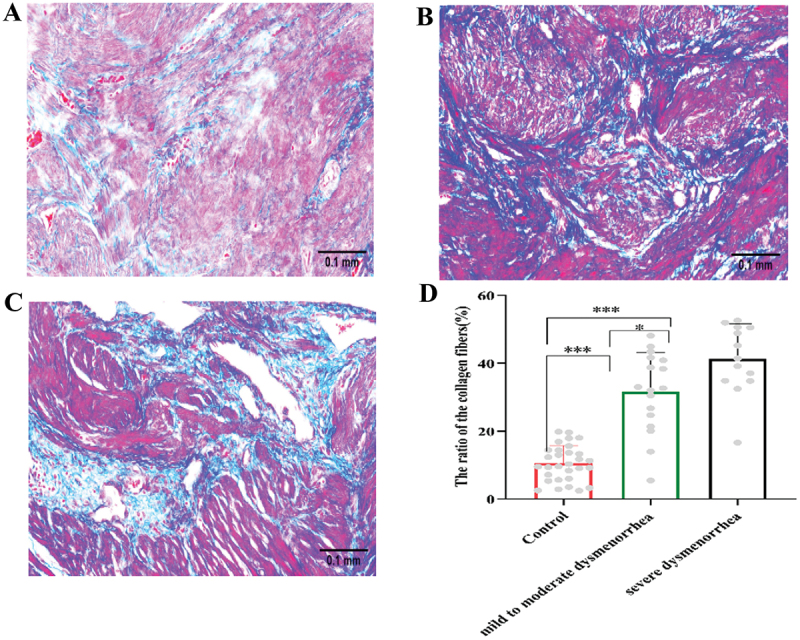
Evidence of fibrosis in adenomyotic myometrium.

Masson’s trichrome staining was performed on both adenomyotic myometrium and control myometrium to detect collagen fibers, a key indicator of fibrosis. In this staining, collagen fibers appear blue, while other structures such as smooth muscle cells are stained red. Control uterine tissues showed minimal fibrotic staining, whereas adenomyotic lesions displayed extensive deposition of collagen fibers (Figures 4(A–C)). The area ratio of collagen fibers was quantitatively assessed using mean optical density measurements. In the myometrium of the AM group, the collagen fiber area ratio was 35.78% ± 11.96%, significantly higher than that in the control group (10.58% ± 5.21%). Both fibrosis indicators demonstrated a statistically significant difference between the two groups (*p* < .05), as summarized in Figure 4D.

(A-C) are representative micrographs (400 ×, scale bar 0.1mm) of collagen fiber staining in each group: (A) the control group; (B) the group of mild to moderate dysmenorrhea; (C) the group with severe dysmenorrhea. (D) Quantitative analysis of the mean area ratio of collagen fiber in each group. * *p* <0.05, ** *p* <0.01, *** *p* < 0.001.

### Correlation of the degree of fibrosis with Gal-1/Gal-3/Gal-9 protein expression

**Figure 5.**
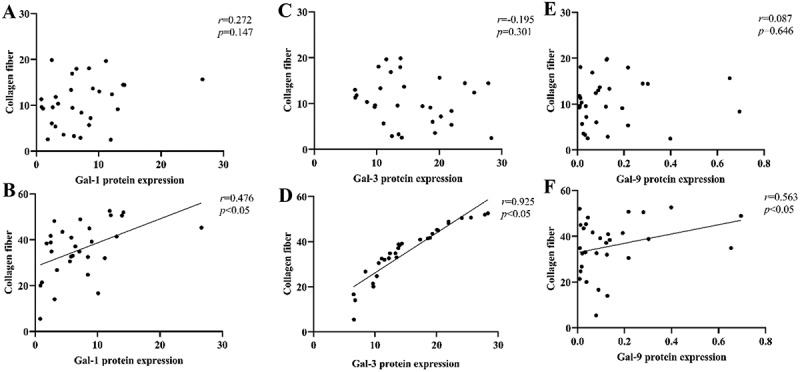
Relationship between the degree of fibrosis and the expressions of gal-1, gal-3, and gal-9.

Spearman correlation analysis revealed that the expression levels of Gal-1, Gal-3, and Gal-9 proteins in the myometrium of the AM group were significantly correlated with the collagen fiber area ratio (*r* = 0.476, *r* = 0.925, and *r* = 0.563, respectively). Among these, Gal-3 expression showed the strongest correlation with the collagen fiber ratio. In contrast, no significant correlation was observed between these variables in the control group (Figure 5).

(A–B) correlation between collagen fiber proportion in the myometrium of control group and AM group with Gal-1 protein expression in the myometrium of the two groups; (C–D) correlation between collagen fiber proportion in the myometrium of control group and AM group with Gal-3 protein expression in the myometrium of the two groups; (E–F) correlation between collagen fiber proportion in the myometrium of control group and AM group, with Gal-9 protein expression in the myometrium of the two groups.

### Correlation of the VAS score with Gal-1/Gal-3/Gal-9 protein expression

**Figure 6.**
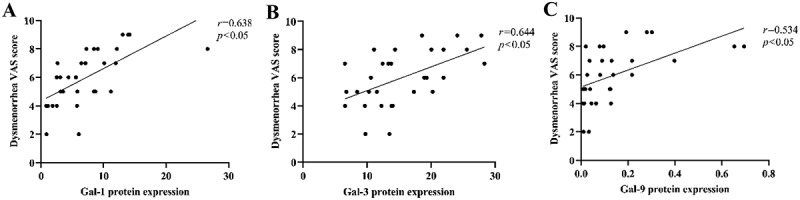
The correlation between protein expression and dysmenorrhea.

Spearman correlation analysis indicated that the expression levels of Gal-1, Gal-3, and Gal-9 proteins in the myometrium of the AM group were significantly correlated with VAS scores (*r* = 0.638, 0.644, and 0.534, respectively), with Gal-3 showing the strongest association. In contrast, no significant correlation was observed between dysmenorrhea severity and the expression of Gal-1, Gal-3, or Gal-9 proteins in the control group (*p* > .05) (Figure 6).

(A–C) respectively represent the correlations between the expressions of Gal-1, Gal-3, and Gal-9 and the VAS score of dysmenorrhea.

### Correlation between the degree of fibrosis and the severity of dysmenorrhea

**Figure 7.**
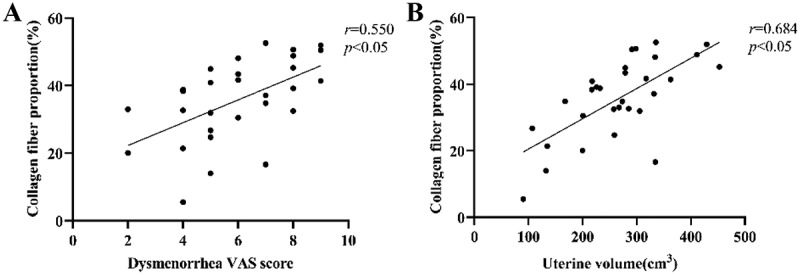
Correlation of collagen fiber with dysmenorrhea severity and uterine volume.

The relationship between fibrosis and dysmenorrhea within the AM group was further examined, as illustrated in Figure 7A. Specifically, the collagen fiber area ratios were found to be 31.59% ± 11.68% in the mild to moderate dysmenorrhea group and 41.27% ± 10.32% in the severe dysmenorrhea group. A Spearman correlation analysis revealed a moderate positive correlation between the collagen fiber area ratio and the severity of dysmenorrhea among patients in the AM group (*r* = 0.550, *p* < .05).

(A) Relationship between collagen fiber proportion and the degree of dysmenorrhea; (B) relationship between collagen fiber proportion and uterine volume in the adenomyosis group.

### Correlation between the degree of fibrosis and uterine volume

The average uterine volume in the AM group (*n* = 30) was 267.64 ± 89.90 cm^3^, significantly larger than that in the control group (81.03 ± 33.29 cm^3^), with a statistically significant difference between the two groups (*p* < .001). Pearson correlation analysis indicated a moderately positive correlation between the myometrial collagen fiber area ratio and uterine volume in the AM group (*r* = 0.684, *p* < .001) (Figure 7B).

## Discussion

Adenomyosis was first formally defined by Bird et al. in 1972 [18]. As a benign gynecological disorder, its etiology remains incompletely understood [19]. It is characterized by progressive development, heterogeneous clinical manifestations, and considerable therapeutic challenges. In the present study, patients with adenomyosis exhibited typical clinical features, including progressive dysmenorrhea, prolonged menstrual bleeding, anemia, elevated serum CA125 levels, and increased uterine volume. These findings are consistent with those reported in previous studies [20]. Further analysis identified the VAS score for dysmenorrhea as a significant predictor of adenomyosis risk. This observation carries notable clinical relevance: as a readily quantifiable parameter in routine practice, the VAS score may serve as a useful tool for screening high-risk populations, thereby enhancing early diagnostic accuracy. Clinicians should maintain a high index of suspicion for adenomyosis in patients presenting with progressively worsening dysmenorrhea or those requiring regular analgesic use, and timely diagnostic evaluation and appropriate intervention should be pursued.

The exact pathogenesis of uterine adenomyosis remains unclear, and fibrosis of the uterine myometrium has emerged as a key research focus. This pathological process arises from an imbalance between chronic inflammation and interstitial remodeling. Persistent local inflammatory stimuliactivate fibroblasts and promote their differentiation into myofibroblasts, leading to excessive deposition and inadequate degradation of extracellular matrix components such as collagen [21]. The fibrotic microenvironment further amplifies inflammatory signals and ultimately leads to myometrial dysfunction and progressive dysmenorrhea, representing a key pathophysiological mechanism of adenomyosis [22].

Gal-1, Gal-3, and Gal-9 are particularly implicated in fibrotic pathogenesis across pulmonary, hepatic, and renal systems [14,23,24]. These proteins exert their profibrotic effects through three principal mechanisms: (1) Modulating immune cell polarization and inflammatory responses, (2) Activating and differentiating of fibroblasts into myofibroblasts, and (3) Promoting ECM deposition and remodeling. Galectins directly/indirectly activate TGF-β (transforming growth factor-β), stimulating fibroblast transformation and synthesis of ECM components (collagen I/III, fibronectin) [25]. Furthermore, via NF-κB activation, they induce sustained production of pro-inflammatory cytokines (IL-6, TNF-α), perpetuating a profibrotic microenvironment [26]. The above evidence suggests a strong role for Gals in the pathogenesis of adenomyosis, potentially through the modulation of myometrial fibrosis. Based on this rationale, we first employed immunohistochemistry to examine the expression and localization of Gal-1, Gal-3, and Gal-9 in myometrial tissue. The results showed that all three proteins were highly expressed in the cytoplasm of myometrial cells from patients with adenomyosis, with expression levels significantly higher than those in the control group. These findings were further validated by Western blotting and real-time quantitative PCR, which demonstrated significant upregulation of both mRNA and protein levels of these galectins in adenomyotic myometrium, confirming their abnormal elevation in this disease. This altered expression pattern is closely associated with smooth muscle hypertrophy, extracellular matrix deposition, and fibrotic progression, further supporting the central regulatory role of the galectin family in gynecological diseases. Moreover, the consistent and significant differential expression of Gal-1, Gal-3, and Gal-9 suggests their potential utility as biomarkers for assessing fibrotic severity and clinical prognosis in adenomyosis.

Given the established functions of Gal-1, Gal-3, and Gal-9 in regulating inflammatory responses, promoting myofibroblast transdifferentiation, and facilitating extracellular matrix deposition, we hypothesize that these galectins play a key role in driving myofibroblast fibrosis in uterine adenomyosis. To test this hypothesis, we sought to quantitatively assess collagen deposition in myometrial tissues using Masson staining. Quantitative analysis revealed a marked and statistically significant increase in the collagen fiber area in the uterine adenomyosis group (35.78% ± 11.96%) relative to the control group (10.58% ± 5.21%, *p* < .05). Correlation analysis revealed that the expression levels of Gal-1, Gal-3, and Gal-9 were each positively correlated with the degree of myometrial fibrosis in uterine adenomyosis. Notably, Gal-3 exhibited the strongest correlation (*r* = 0.925, *p* < .05). These findings suggest a synergistic role for Gal-1, Gal-3, and Gal-9 in promoting fibrosis in uterine adenomyosis, with Gal-3 potentially serving as a core regulator. This galectin trio may drive uterine remodeling and sclerosis by modulating collagen deposition and fibroblast activation, thereby contributing to disease progression. Together, these findings provide experimental evidence for the mechanisms underlying myometrial fibrosis in uterine adenomyosis and offer potential therapeutic targets for anti-fibrotic treatment.

Previous studies and our preliminary work have demonstrated that Gal‑1, Gal‑3, and Gal‑9 contribute to the fibrotic process in the uterine myometrium by promoting collagen deposition, inducing fibroblast activation, and accelerating extracellular matrix remodeling. Fibrosis represents both the core pathological feature of uterine adenomyosis and the structural basis for the progressive worsening of dysmenorrhea [27]. In this context, an increase in the VAS score for dysmenorrhea may serve as a clinical surrogate for the progression of fibrosis. Building on this premise, our study sought to analyze the relationship between the expression of these three molecules and both the VAS score for dysmenorrhea and the degree of myometrial fibrosis, using these parameters as key indicators of disease progression. The results demonstrated that the expression levels of Gal-1, Gal-3, and Gal-9 were significantly positively correlated with the VAS score for dysmenorrhea, and that the VAS score itself was closely associated with the degree of myometrial fibrosis. These findings suggest an intrinsic link between molecular abnormalities, pathological changes, and clinical symptoms. This study verified the pro-fibrotic effects of Gal‑1, Gal‑3, and Gal‑9 in adenomyosis and is the first to clinically confirm their association with core symptoms. Future targeted interventions targeting these molecules may simultaneously reverse fibrosis and relieve dysmenorrhea, offering novel strategies for the clinical management of adenomyosis.

This study systematically examined the expression of Gal‑1, Gal‑3, and Gal‑9 in the myometrium of patients with adenomyosis, determined their correlations with the degree of fibrosis and the severity of dysmenorrhea, and provided experimental evidence for elucidating the molecular mechanisms underlying fibrosis in adenomyosis. Nevertheless, this study has several limitations. First, as a single-center observational study with a relatively limited sample size, the findings require validation through larger, multi-center studies to enhance their generalizability. Second, although Gal-3 was identified as the molecule most closely correlated with disease fibrosis and dysmenorrhea – highlighting its potential as a key therapeutic target – its functional role has yet to be validated through gene knockout, overexpression, or inhibitor studies in cell and animal models. More broadly, the expression correlations of all three galectins were only established at the tissue level, and the specific molecular mechanisms and signaling pathways involved remain to be elucidated. Additionally, the lack of long-term prognostic follow-up in this study precluded analysis of the relationship between galectin expression and disease progression, symptom recurrence, or therapeutic efficacy. Therefore, their clinical utility as biomarkers for disease monitoring and prognostic assessment warrants further investigation.

In summary, this study demonstrates that Gal-1, Gal-3, and Gal-9 are abnormally overexpressed in the myometrial tissues of uterine adenomyosis and are significantly positively correlated with both the degree of fibrosis and the severity of dysmenorrhea. These findings suggest that these three galectins may synergistically regulate fibrotic processes, thereby contributing to disease pathogenesis and the development of dysmenorrhea. This study provides potential biomarkers for disease assessment and dysmenorrhea prediction, as well as a theoretical foundation for targeted therapeutic strategies. Nevertheless, the precise mechanisms and clinical utility of these galectins warrant further validation through large-scale, multi-center studies and functional experiments.

## Conclusion

In conclusion, the upregulation of Gal-1, −3, and −9 in adenomyosis correlates positively with the severity of myometrial fibrosis and dysmenorrhea. This suggests a mechanism where these galectins promote collagen deposition, thereby driving fibrotic changes that exacerbate pain, establishing them as promising diagnostic biomarkers and therapeutic targets.

## Clinical perspectives

• AM can cause progressively aggravated dysmenorrhea in patients and affect their quality of life. The expressions of Gal-1, Gal-3, and Gal-9 in the myometrium of AM patients are increased.

• In clinical study samples, Gal-1, Gal-3, and Gal-9 expression in the myometrium tissue correlated with the menstrual length, VAS score for dysmenorrhea, hemoglobin, CA125, and uterine volume of AM patients. Furthermore, Gal-1, Gal-3, and Gal-9 can all promote myometrial fibrosis in patients with adenomyosis.

• Given the lack of effective pharmacological treatment for AM, our study confirmed that targeting Gal-1, Gal-3, and Gal-9 can offer new hope for the treatment.

## Data Availability

The data supporting this study’s findings are available from the corresponding author upon reasonable request.

## Abbreviations

AM: adenomyosis
Gal-1: galectin-1
Gal-3: galectin-3
Gal-9: galectin-9
EMT: epithelial-mesenchymal transition
CIN II/ III: cervical intraepithelial neoplasm II/ III
IUD: intrauterine device
BSA: bovine serum albumin
PBS: phosphate-buffered saline
BMI: body Mass Index
VAS: visual analogue score
ORs: odds ratios
CIs: confidence intervals
TGF-β: transforming growth factor-β
ECM: extracellular matrix
IL-6: Interleukin-6
TNF-α: Tumor Necrosis Factor-alpha
